# Characterisation of early metazoan secretion through associated signal peptidase complex subunits, prohormone convertases and carboxypeptidases of the marine sponge (*Amphimedon queenslandica*)

**DOI:** 10.1371/journal.pone.0225227

**Published:** 2019-11-12

**Authors:** Michael J. Hammond, Tianfang Wang, Scott F. Cummins

**Affiliations:** Genecology Research Centre, University of the Sunshine Coast, Maroochydore Dc, Queensland, Australia; Biocenter, Universität Würzburg, GERMANY

## Abstract

Efficient communication between cells requires the ability to process precursor proteins into their mature and biologically active forms, prior to secretion into the extracellular space. Eukaryotic cells achieve this via a suite of enzymes that involve a signal peptidase complex, prohormone convertases and carboxypeptidases. Using genome and transcriptome data of the demosponge *Amphimedon queenslandica*, a universal ancestor to metazoan multicellularity, we endeavour to bridge the evolution of precursor processing machinery from single-celled eukaryotic ancestors through to the complex multicellular organisms that compromise Metazoa. The precursor processing repertoire as defined in this study of *A*. *queenslandica* consists of 3 defined signal peptidase subunits, 6 prohormone convertases and 1 carboxypeptidase, with 2 putative duplicates identified for signal peptidase complex subunits. Analysis of their gene expression levels throughout the sponge development enabled us to predict levels of activity. Some *A*. *queenslandica* precursor processing components belong to established functional clades while others were identified as having novel, yet to be discovered roles. These findings have clarified the presence of precursor processing machinery in the poriferans, showing the necessary machinery for the removal of precursor sequences, a critical post-translational modification required by multicellular organisms, and further sets a foundation towards understanding the molecular mechanism for ancient protein processing.

## Introduction

The ability for cells to coordinate as a multicellular organism represents one of the most important developments in the evolutionary timeline of life [1, 2]. Such collaboration required cell communication, the ability for cells to both stimulate and inhibit neighbouring or distant cells via messenger molecules to undertake a variety of metabolic, developmental and electrical processes. These include signalling for cell growth, development and specialisation, the ability to induce programmed cell death and allorecognition [3]. Cell communication in turn required the development of a secretory pathway; a means in which cells can secrete various hormones, proteins and peptides into extracellular space. Cell secretion occurs in two different patterns, either in a constant process known as constitutive secretion, or in a regulated manner, dependent on signals before extracellular release of contents [4].

Bioactive proteins that are secreted from cells originate from precursor sequences that consist of additional regions that may also fulfil various functions while a protein is in transit that, however, must ultimately be removed for the protein to become functional. Additional sequences typically include an N-terminal signal sequence or peptide, often termed the “pre” region. This serves as a barcode to ensure the protein directed into the secretory pathway, from the ribosome of synthesis to the endoplasmic reticulum [5]. Here, the pre-region is removed by a signal peptidase complex (SPC), which is composed of a variety of subunits referred to as signal peptidases (SP) [6]. From the endoplasmic reticulum the protein is transported to the Golgi apparatus, where the protein is packaged into secretory vesicles, for transport to the cell membrane. Proteins may also have an additional “pro” region attached either to the N- or C-terminus. The pro-region can serve a variety of functions, such as protecting the mature region (sequence sans ‘pre’ and ‘pro’ region) from degradation, preventing premature protein activation and assisting in correct folding of the mature region [7]. As the protein is being packaged and secreted, this pro region is removed by a prohormone convertase (PC) [4]. C-terminus removal results in the recognition site still being attached to the mature protein, which then must be removed by a carboxypeptidase (CP) before full functionality can be achieved [8].

Although cell secretion is an essential component for all metazoans, the process first evolved in single-celled organisms who also had requirements for limited cell secretion; for example, to protect and to communicate within a species [6]. We now know that in humans there are 5 SPs, 7 basic residue cleaving PCs and 2 CPs that are involved in processing secreted proteins [4, 6, 9].

Of these 3 families, PCs, which are related to the bacterial ‘subtilisin’ [10] have shown the greatest diversification through the evolution of multicellularity. While 4 to 5 SPs, and 1 to 2 CPs are typically been found in a range of both unicellular and multicellular organisms [11–13], PCs show varied distribution across the tree of life, with yeast *Saccharomyces cerevisiae* species possessing 1 (Kexin) [4], *D*. *melanogaster* having 3 PCs, 2 of which being distinct variants of the same convertase ‘Furin’ [14], while *Caenorhabditis elegans* possesses 5, 2 of which (blisterase and aex-5) are nematode specific, with blisterase generating 9 isoforms [15]. Similarly, the SPs, PCs and CPs of other metazoa have been determined or predicted. What is not known however, is the state of preprotein processing peptidases at the advent of metazoan development, during and succeeding the transition from single-celled organisms to multicellular metazoa.

Marine sponges represent one of the oldest extant members of metazoa [16]. Despite having evolved over 600 million years ago, sponges have remained relatively unchanged, occupying a unique ecological niche as immobile filter feeders in oceans and freshwater systems [17]. Sponges have simple body designs, lacking nervous systems, true tissue types or even body symmetry in most cases, yet they engage in the requisite signalling and cell communication activities which qualify them as true members of metazoa [18]. As such, they represent the closest link to the first metazoan species to have developed, which would have most likely resembled sponges. The sequencing of the genome and transcriptome for sponge species *Amphimedon queenslandica* enables us to analyse the closest available link to the advent of metazoa, bridging the transition from single celled organisms to metazoan multicellularity in terms of SPs, PCs and CPs in relevant species [3]. We also have the opportunity to assess the secretory capabilities of *A*. *queenslandica* (as well as sponges in general), and to determine the essential SPs, PCs and CPs needed for the development of metazoa.

Here, we report the identification, phylogeny and developmental expression pattern of *A*. *queenslandica* SP, PC and CP genes, which are likely to be involved in processing and cleaving precursor proteins. We identify the peculiar absence of components such as SP 22/23, as well as the unusual size of certain PCs and CPD, suggesting developments that are either sponge-specific or confined to certain lower metazoan members.

## Methods

### Identification of protein secretory machinery sequences

The University of Queensland granted permission for the collection of sponge specimens and their return on Heron Island Research Station

Well established protein sequences corresponding to *Homo sapiens*, *Drosophila melanogaster*, *Danio rerio and Caenorhabditis elegans (*SPCs, PCs and CPs) were collected from the NCBI database. SP, PC and CPs proteins of other organisms were compiled for comparison using Position-Specific iterated BLASTp searches against *H*. *sapiens* SP, PC and CP families, refined in further iterations using established sequences of *D*. *rerio*, *C*. *elegans* and *D*. *melanogaster*. Searches were restricted to species that allowed for an even phylogenetic distribution of metazoa, as well as select pre-metazoan choanoflagellates and basal protists.

SPs, PCs and CPs obtained from other species of NCBI included: *Dictyostelium purpureum*, *Monosiga brevicollis*, *Salpingoeca rosetta*, *Trichoplax adhaerens*, *Nematostella vectensis*, *Hydra vulgaris*, *Strongylocentrotus purpuratus*, *Lottia gigantea*, *Tribolium castaneum*, *Capsaspora owczarzaki* and *Chlamydomonas reinhardtii*. For simplicity, if isoforms were present, only one was used for comparison. S1 File lists all sequences and accession IDs.

*H*. *sapiens* sequences of SPC, PC and CP were also blasted against members of ctenophora, including *Pleurobrachia bachei* transcriptome [19] accessed at Neurobase (Filtered gene models) as well as *Mnemiopsis leidyi* genome portal project (Protein Models 2.2) [20]. Cutoff values of 1x10^-70^ were used for *P*. *bachei* and 1x10^-150^ for *M*. *leidyi*. Defined PCs and CPs from basal eukaryote *Chlamydomonas reinhardtii* [13] were taken from MEROPS peptidase database v12.1 [21].

The complete proteome dataset of *A*. *queenslandica* was obtained from the Ensemble Metazoa database (http://www.ensembl.org/index.html). *A*. *queenslandica* SP, PC and CP proteins were searched for in this dataset using HMMER package v3.1 [22] against clustalW aligned proteins generated by Mega10.0.5 [23] of *H*. *sapiens*, *D*. *rerio*, *D*. *melanogaster* and *C*. *elegans*, using an E-value cut-off of 1x10^-25^ for SPC and 1x10^-50^ for PC and CP.

### Comparative protein and gene expression analysis

Protein amino acid sequences were aligned via multiple sequence alignments using MEGA10.0.5 [23], and presentation was prepared via MiKTeX TEXshade [24]. PC sequences were accepted if catalytic region contained critical D, H and S residues (S1 Fig). Using MEGA10.0.5, maximum likelihood trees were constructed for SPC, PC and CP sequences, using default parameters and pairwise deletion, with site cut-off coverage of 95% [23]. Bootstrap test of phylogeny was employed for all phylogenetic trees, using 500 replicates [25].

Deduced sponge protein sequences were analysed via SignalP, TMHMM and Simple Modular Architecture Research Tool (SMART) [26–28], prior to schematic representation using the Domain Graph illustrator DOG 2.0 [29]. Topology presentation was prepared for highest matches to SP components with greatest length, using the MiKTeX TEXtopo [24]. Gene expression levels for *A*. *queenslandica* SPs, PCs and CPs transcripts were obtained from CEL-Seq 2.1 data reported in Hashimshony et al [30]. Replicate expression values for each stage were averaged, generating 25 stages within *A*. *queenslandica’s* life cycle.

## Results and discussion

### Signal peptidase complexes (SPCs)

**Fig 1:**
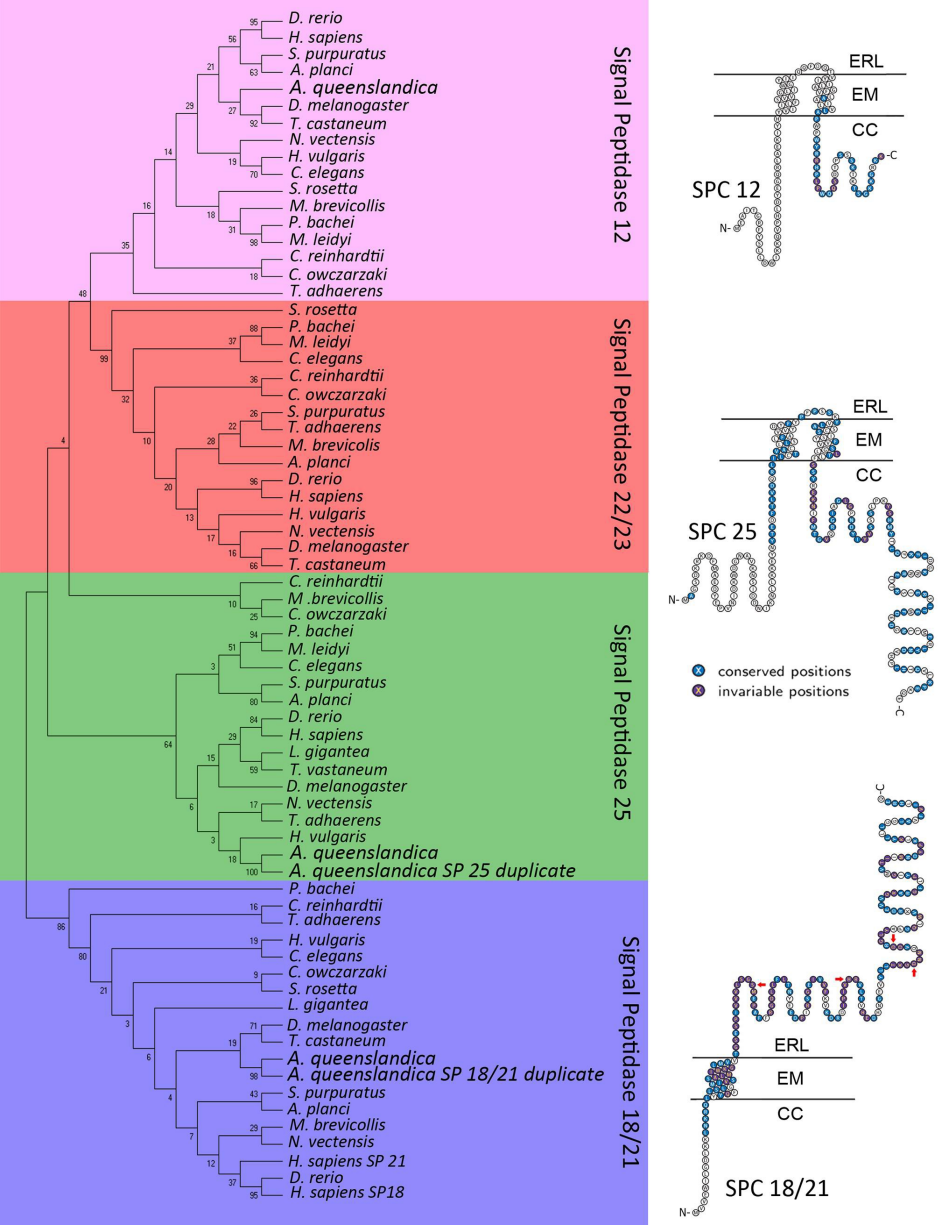
Phylogeny of the signal peptidase complex family. Tree was constructed using Maximum Likelihood method and JTT matrix-based model. The bootstrap consensus tree was inferred from 500 replicates and is taken to represent the evolutionary history of the taxa analysed. Branches corresponding to partitions reproduced in less than 50% bootstrap replicates were collapsed. The percentage of replicate trees in which the associated taxa clustered together in the bootstrap test (500 replicates) are shown next to the branches. Initial trees for heuristic search were obtained by Neighbour-Join and BioNJ logarithms to a matrix of pairwise distances estimated using JTT model and then selecting topology with superior likelihood. This analysis involved 70 amino acid sequences. All positions with less than 95% site coverage were eliminated. A total of 72 positions were used in the final dataset. Different colours used to distinguish signal peptidase subunits (SP12: Pink, SP22/23: Red, SP25: Green, SP18/21: Blue). Accession numbers for all species sequences is shown in S1 File. Right: Topology schematics for *A*. *queenslandica* SPs generated for highest scoring hit, showing conservation between species. Red arrows on SP 18/21 indicate conserved residues essential for catalytic function. ERL, endoplasmic reticulum lumen; EM, endoplasmic membrane; CC, cell cytoplasm. Accession numbers for all species sequences is shown in S1 File.

*In silico* screening of the *A*. *queenslandica* genome identified 5 sequences matching to SPC subunits. To examine the relationships between *A*. *queenslandica* and other phyla SPs, we constructed a phylogenetic tree (Fig 1). From this analysis we can define one ortholog for SPC12 and two orthologs for SPC18/21 and SPC25, with high bootstrap support. Sequence alignments of both orthologues for SPC18/21 and SPC25 show almost identical sequence conservation, suggesting that duplication events have taken place here. In both cases we termed the longer sequence, which showed a higher hit score, as the original sequence, and the shorter with a lower E value as the duplicate (S1 File). Interestingly, mammal’s contain two homologues to SPC18/ 21, referred to as SPC18 and SPC21 based on protein size, both of which are weakly homologous to bacterial SPC1, though this expansion is confined to mammals and thus unrelated to *A*. *queenslandica* [31]. Both SP 18 and 21 function by removing the signal peptide from precursor proteins destined for the secretory pathway [5].

Both sponge orthologues to SPC18/21 displays amino acid conservation at S_56_ and H_96_, as well as D_116_ and D_122_ (Fig 1), all of which are essential for catalytic activity in eukaryotic SPCs [5]. We may therefore assume that these sponge SPC18/21s are functional. A single transmembrane domain is predicted within the N-terminal region, while the proceeding sequence, including the catalytic region, is embedded within the endoplasmic lumen. This structural arrangement is consistent with the known topology of functional SPC18/21 [6]. By contrast, *A*. *queenslandica* SP12 and both SP25s have two transmembrane domains with both their N- and C-terminal sequences extending out into the cell cytoplasm while a short connecting loop is all that is present in the endoplasmic reticulum lumen (Fig 1). These SPs are not known to be involved in protein cleavage, and their roles in *A*. *queenslandica*, as well as other eukaryotic organisms, are less certain [32]. Studies in the yeast *Saccharomyces cerevisiae* have shown that SP12 plays a role in increasing the cleaving efficiency of SP18/21 [33]. Similar studies conducted on SP25 have shown it to interact with protein translocator Sec61 to facilitate movement of signal peptide bound proteins across the endoplasmic reticulum for processing by SPC18/21 [34]. Until studies are performed to assess the function of sponge SP12 and 25, we can only speculate that these SPs are performing similar support roles to those found in *S*. *cerevisiae*, to increase cleavage efficiency and preprotein translocation.

Interestingly, *A*. *queenslandica* does not appear to possess an ortholog for SP22/23, whereas SP22/23 has been identified in the choanoflagellate species, *M*. *brevicollis* and *S*. *rosetta* and single celled eukaryotes *C*. *reinhardtii*, *D*. *purpureum* and *C*. *owczarzaki* (Fig 1). Although lost in *A*. *queenslandica*, it is present in many other more complex eumetazoan species, such as *C*. *elegans*, *D*. *melanogaster* and *H*. *sapiens* (Fig 1). The role that SP22/23 plays in eukaryotic organisms is also yet to be determined, although it is not thought to be a ‘cleaving’ SP [5]. *T*. *adhaerens*, one of the only other extant animal phyla not classed as eumetazoa, also does not display SP22/23. Further analysis of other sponge species may help to confirm the absence of SP22/23 in the poriferans.

**Fig 2:**
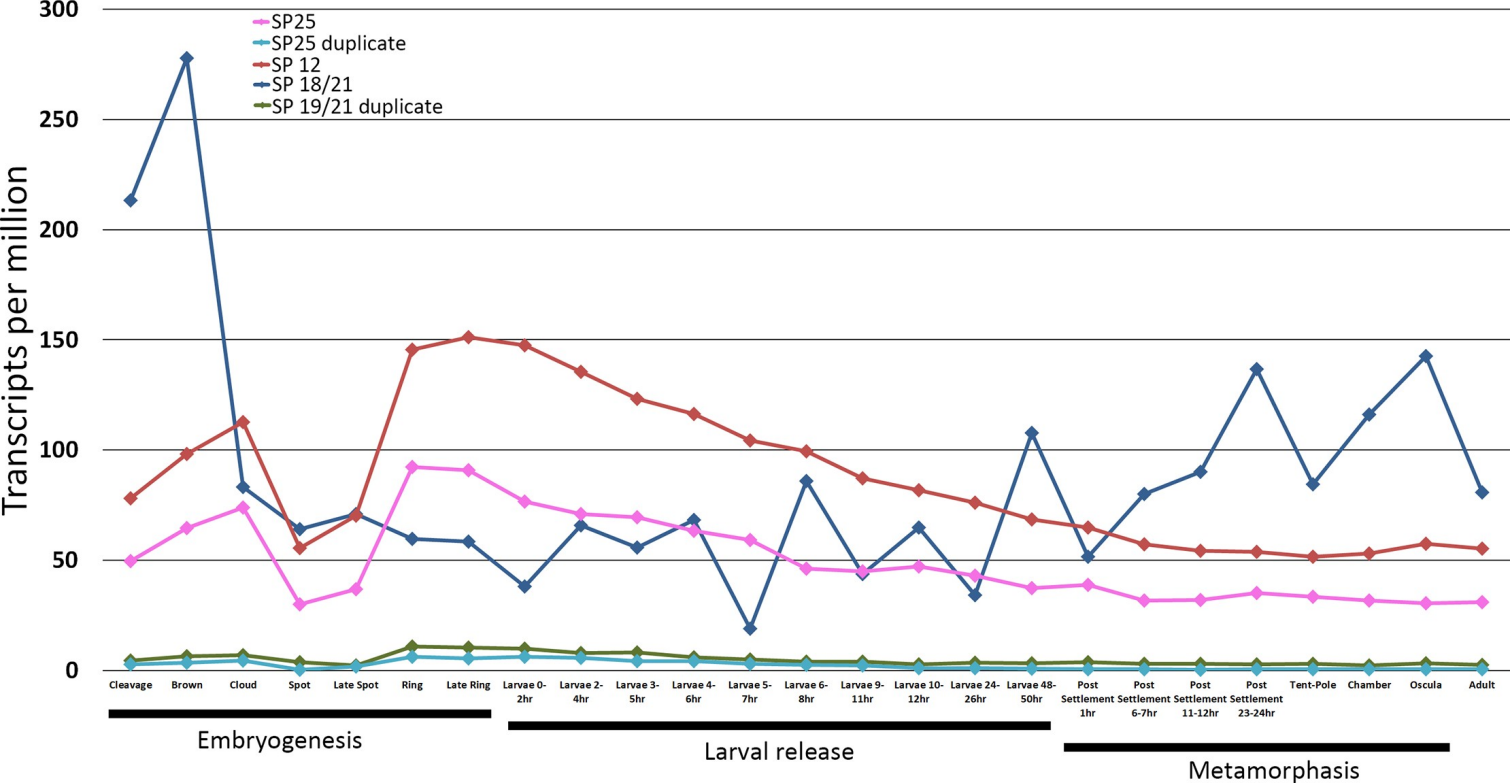
Gene expression of signal peptidase complex subunits throughout life-stages of *A*. *queenslandica*. Stages are additionally grouped into 4 main phases; Embryogenesis, Larval development, Metamorphosis and Adult.

We investigated the temporal expression the *SPs* during *A*. *queenslandica* embryonic and larval development to metamorphosis and adult. *SP18/21*, shows highest expression at the ‘brown’ stage of embryogenesis, before dramatically dropping at ‘cloud’ stage, then generally increasing throughout larval and metamorphosis phases (Fig 2). By comparison, *SP12* and *SP25* shows peak expression at ‘late ring’ and ‘ring’ stage respectively, after which generally decreasing throughout larval, metamorphosis and adult phases of life (Fig 2). Putative duplicates of *SP18/21* and *SP25* show minimal expression throughout all life phases, with no notable changes which suggest that these serve as dispensable sequences, presumably being filled by *SP18/21* and *SP25* (Fig 2). The expression patterns of all signal peptidase subunits which all peak during embryogenesis (Fig 2), suggest that secretion requirements are highest at this point, a period that requires growth, development and structural reorganisation of various cells [35, 36].

### Prohormone convertases (PCs)

**Fig 3:**
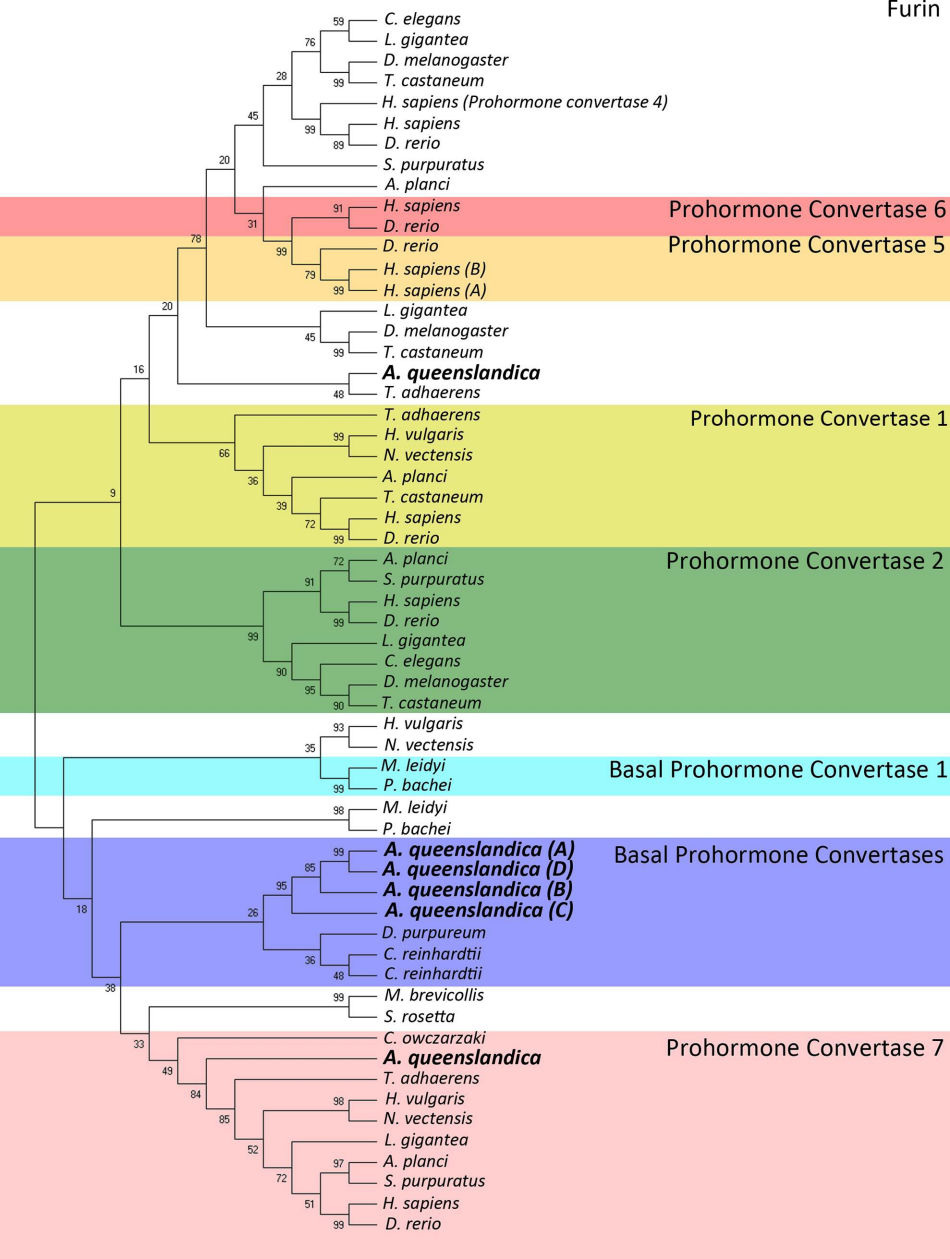
Phylogeny of the prohormone convertase family. Tree was constructed using Maximum Likelihood method and JTT-matrix based model. The bootstrap consensus tree was inferred from 500 replicates is taken to represent the evolutionary history of the taxa analysed. Branches corresponding to partitions reproduced in less than 50% bootstrap replicates were collapsed. The percentage of replicate trees in which the associated taxa clustered together in the bootstrap test (500 replicates) are shown next to the branches. Initial trees for heuristic search were obtained by Neighbour-Join and BioNJ logarithms to a matrix of pairwise distances estimated using JTT model and then selecting topology with superior likelihood. This analysis involved 59 amino acid sequences. All positions with less than 95% site coverage were eliminated. A total of 459 positions were used in the final dataset. Different colours used to distinguish signal peptidase subunits (White: furin, Red: prohormone convertase 6, Orange: prohormone convertase 5, Yellow: prohormone convertase 1, Green: prohormone convertase 2, Cyan: basal prohormone convertase 1, Blue: basal prohormone convertases, Pink: prohormone convertase 7). Accession numbers for all species sequences is shown in S1 File.

*In silico* screening of the *A*. *queenslandica* genome initially identified 12 PCs (S1 Fig). Analysis of catalytic domains showed that only 6 possessed all catalytic triad of amino acids (i.e. D-H-S) (S1 Fig), which were considered for further analysis. While 10 putative PCs were previously identified in *A*. *queenslandica* [3], analysis of critical residues present in later genome editions shows that only 6 functional PC sequences exist in *A*. *queenslandica*. Mammalian genomes encode 7 basic peptide cleaving PCs [37], while *C*. *elegans* encodes 5 [15], *D*. *melanogaster* 3 [14] and *S*. *cerevisiae* 1 [4]. Phylogenetic analysis of *A*. *queenslandica* PC-like sequences with other phyla PCs could define one ortholog for PC7, while four PC-like paralogous proteins form a separate clade of novel PCs (NPCs) A-D with other, more basal PCs. Additionally, one *A*. *queenslandica* sequence localises within a cluster of furin sequences, hence we designate this as *A*. *queenslandica* furin (Fig 3).

**Fig 4:**
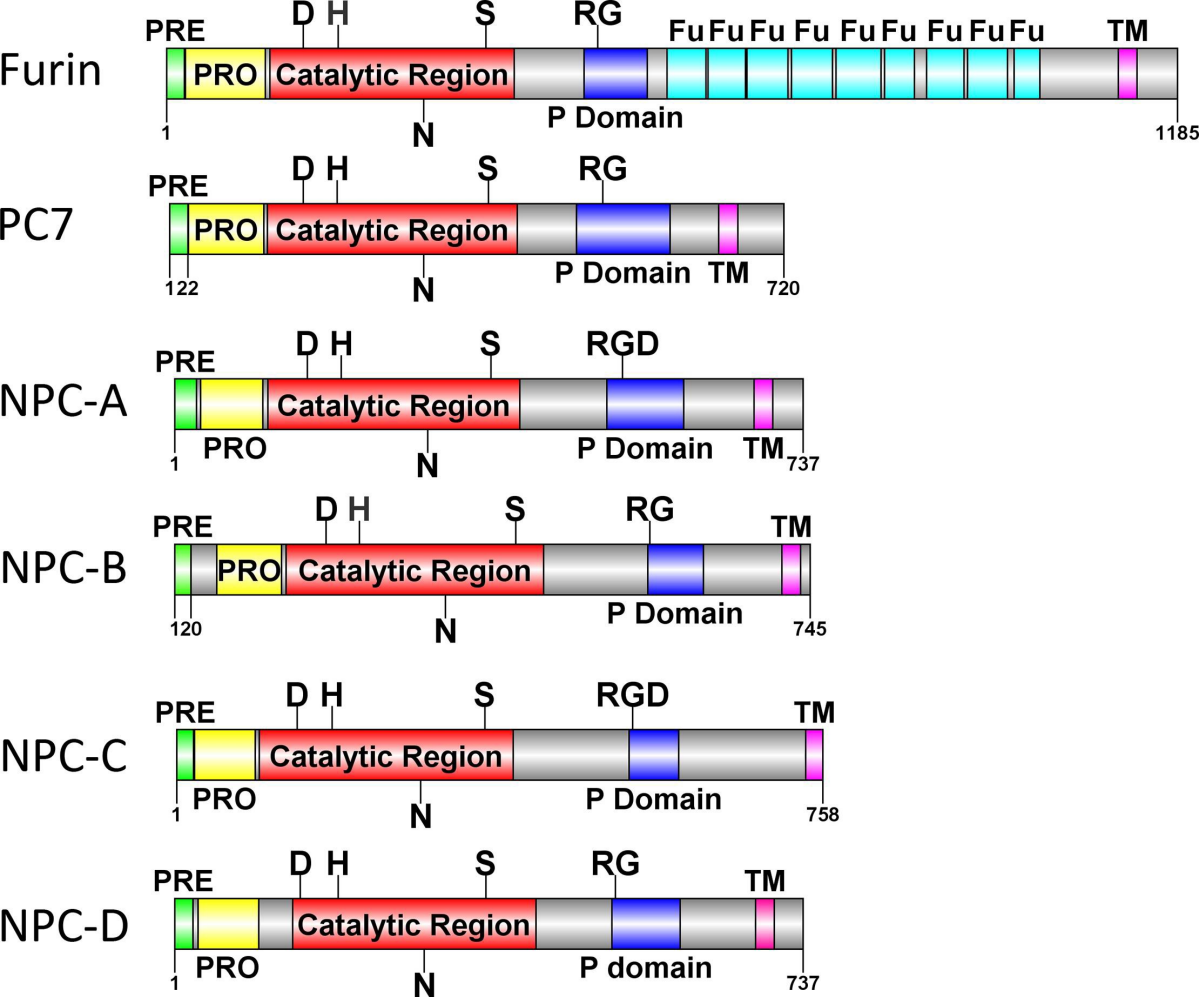
Schematics showing organisation of *A*. *queenslandica* PCs. Schematics show predicted ‘PRE’ and ‘PRO’ regions, transmembrane domains (TM), catalytic regions with essential amino residues (D, H, S), P domains with the presence of important motif (RG or RGD) and Furin-like regions (Fu).

Analysis of the catalytic region of this furin shows the presence of an asparagine (N) at residue 302 (Fig 4). This differentiates it from PC2, where this residue is substituted for aspartic acid (D), [38], which is critical for its activation by protein 7B2 [39]. Similarly, all other *A*. *queenslandica* PCs show the presence of N in this position of the catalytic region (Fig 4 and S1 Fig). While previous phylogenetic analysis predicted the presence of 5 PC2-like convertases [3] analysis of catalytic domains, along with the absence of activating protein 7B2 (also searched for via Hidden Markov Models), suggests that PC2 is in fact absent from the *A*. *queenslandica* genome. While an RG motif is present in the P domain of *A*. *queenslandica* furin (Fig 4), a feature essential for functional activity of PCs [4], the lack of the succeeding D residue, creating the typical ‘RGD’ motif also shows that this PC does not belong to the PC1 family. In organisms that contain PC1 and/or PC2, the RGD motif is essential in ensuring that PC1 and PC2 are sorted to the regulated secretory pathway [40]. Interestingly, the RGD motif is present in Novel PCs A and C (Fig 4). Additionally, the presence of transmembrane domains in all *A*. *queenslandica* PCs, a feature not found in neuroendocrine PCs [4], suggests the absence of the PC1 family in this sponge species. Importantly, the presence of numerous cysteine rich furin-like repeats between the P-domain and transmembrane region suggests the true identity of this PC, hence we designate this PC ‘furin’. Like *T*. *adhaerens* furin, this A. *queenslandica* PC demonstrates extensive furin rich repeats (Fig 4), and exhibits a mature length of over 1000 amino acids, whereas typical PCs range from 700–800 residues [4].

**Fig 5:**
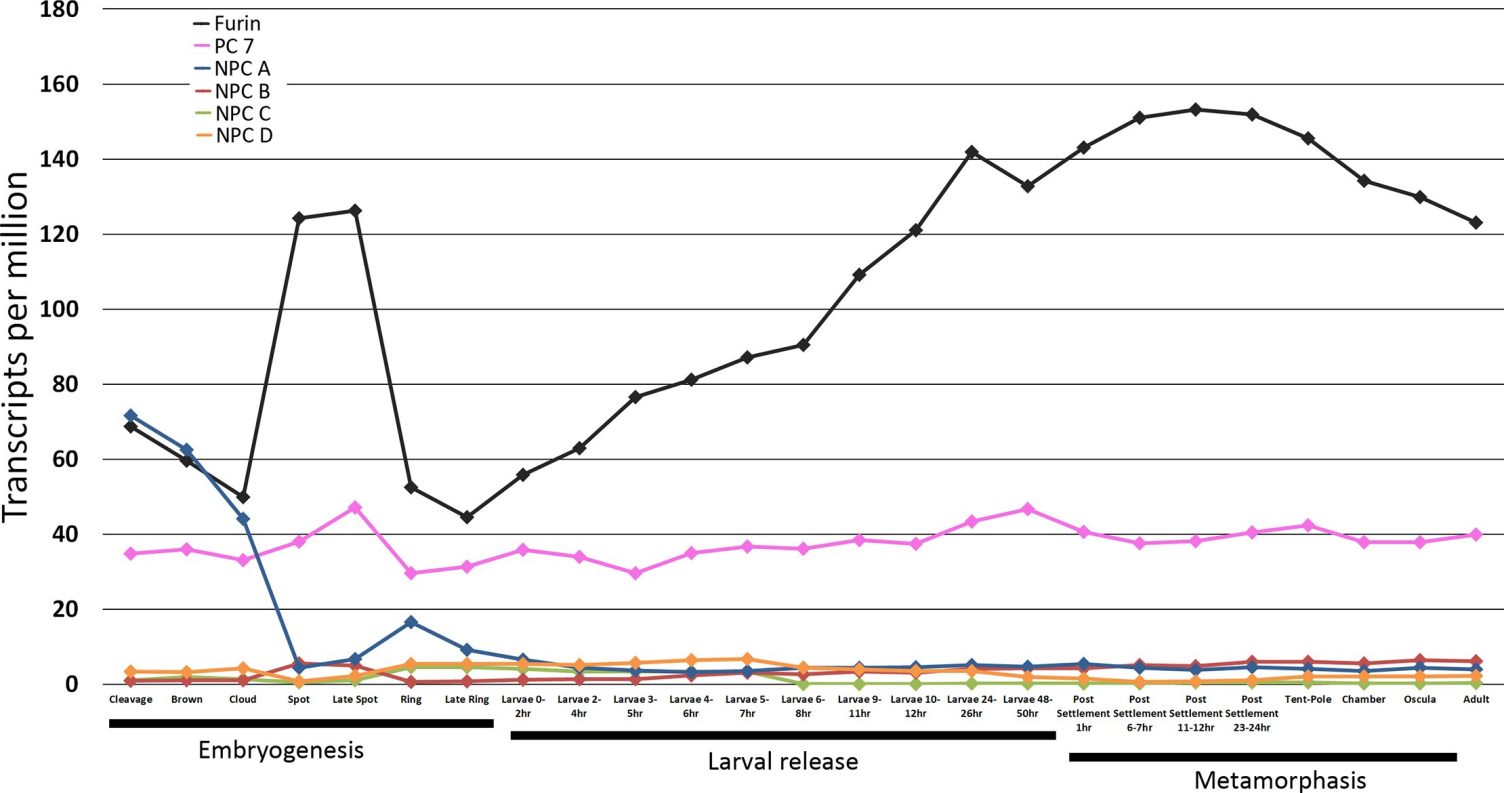
Gene expression of prohormone convertases throughout life-stages of *A*. *queenslandica*. Stages are additionally grouped into 4 main phases; Embryo, Larvae, Metamorphosis and Adult.

Gene expression analysis of *A*. *queenslandica PCs* during development, demonstrates that defined PCs of *furin* and *PC7* show the highest relative expression levels throughout the sponge’s life cycle (Fig 5). *Furin* shows the highest sustained expression, peaking at 11–12 h post settlement during metamorphosis. *PC7* expression shows fairly consistent expression by contrast, peaking during late spot stage of embryogenesis (Fig 5). Of the 4 *NPCs*, *NPC-A* shows the highest relative expression levels throughout development, being highest at the cleavage stage of embryogenesis (Fig 5). Expression of the other 3 novel *NPCs* is minimal and undynamic, which suggests that these PCs may be functionally redundant (Fig 5).

### Carboxypeptidases (CPs)

**Fig 6:**
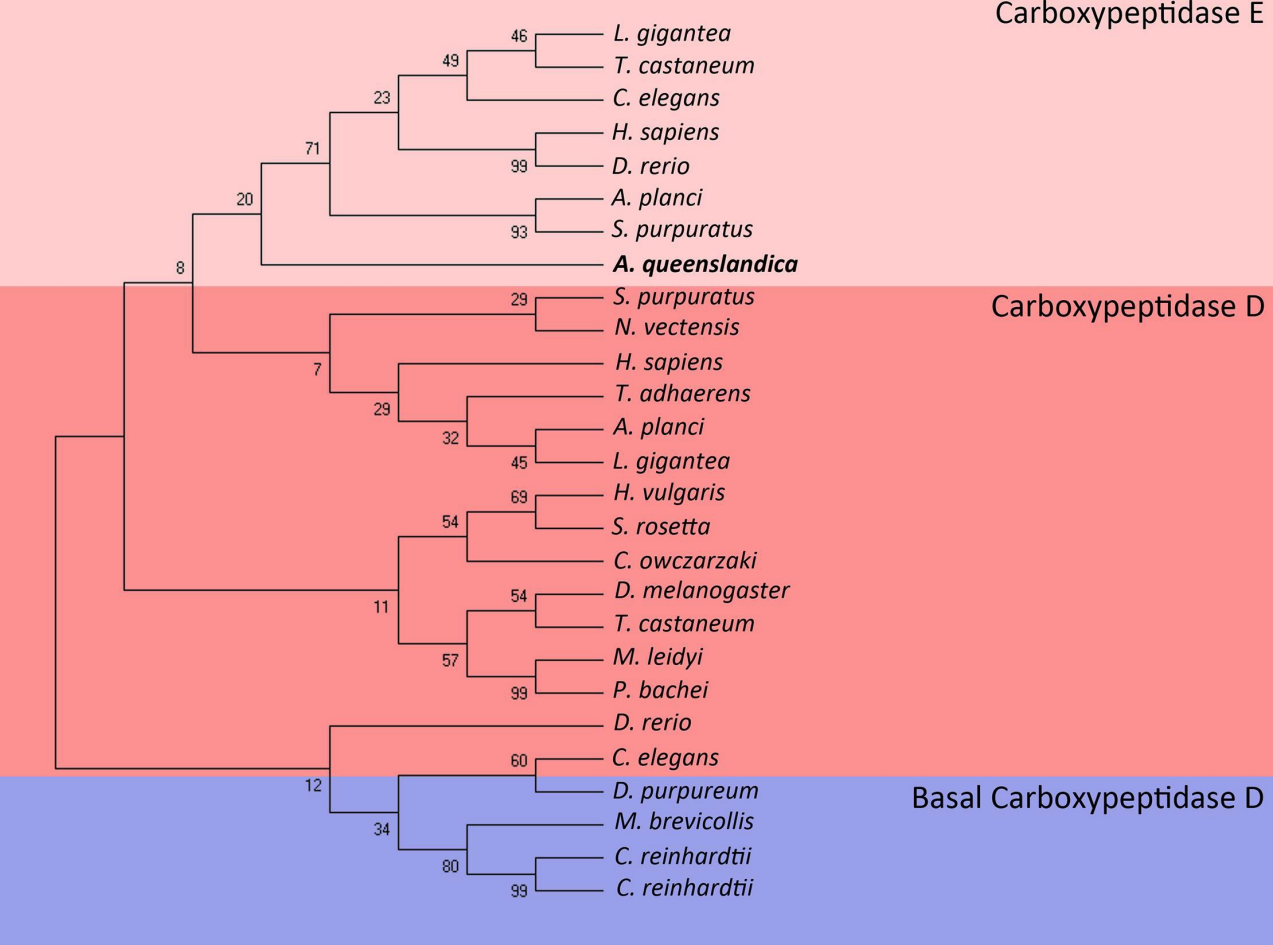
Phylogeny of the carboxypeptidase family. Tree was constructed using Maximum Likelihood method and JTT-matrix based model. The bootstrap consensus tree was inferred from 500 replicates is taken to represent the evolutionary history of the taxa analysed. Branches corresponding to partitions reproduced in less than 50% bootstrap replicates were collapsed. The percentage of replicate trees in which the associated taxa clustered together in the bootstrap test (500 replicates) are shown next to the branches. Initial trees for heuristic search were obtained by Neighbour-Join and BioNJ logarithms to a matrix of pairwise distances estimated using JTT model and then selecting topology with superior likelihood. This analysis involved 27 amino acid sequences. All positions with less than 95% site coverage were eliminated. A total of 313 positions were used in the final dataset. Different colours used to distinguish signal peptidase subunits (Pink: carboxypeptidase E, Red: carboxypeptidase D, Blue: basal carboxypeptidase D). Accession numbers for all species sequences is shown in S1 File.

While a number of CPs exist with varied functions including protein digestion and catabolism, for the purpose of this article we have restricted our focus to CPs only involved in the maturation of proteins [41]. *In silico* screening of the *A*. *queenslandica* genome identified 1 CP above the accepted threshold. Phylogenetic analysis *A*. *queenslandica* CP with other phyla CPs showed distribution closer to carboxypeptidase E (CPE) than with the carboxypeptidase D (CPD) family (Fig 6). In mammals, CPD functions as a secondary neuropeptide processor, in association with prominent neuropeptide processor CPE, which is found exclusively in neuroendocrine cells [42]. Mice that do not express a functional CPE do not die from lack of proper neuropeptide processing since the CPD can fill this functional role, though not perfectly; as mice still display phenotypes of reduced insulin and other neuropeptide processing defects, showing CPEs neuropeptide-cleaving preferences [42]. In some non-mammalian organisms such as *D*. *melanogaster* however, only CPD is present, which has shown to be capable of neuropeptide cleavage [12]. *D*. *melanogaster* possess multiple splice forms of CPD, some of which localise to regulated secretory vesicles, and enable this variant of CPD to mimic the distribution of CPE [43].

While phylogenetic distribution suggests that this *Amphimedon* sequence belongs to the CPE clade, the fact the *Amphimedon* CP contains a transmembrane domain (which is exclusive to CPD) [44], the narrower phylogenetic distribution of CPE when compared to CPD, as well as the superior matching score to CPD over CPE, generated from hidden markov model searches against the *A*. *queenslandica* genome, we designate this sequence as belonging to CPD, which is in agreement with previous predictions of carboxypeptidases in *A*. *queenslandica* [3]. This sponge CPD protein does contain some differences compared with other known CPDs; notably, it is predicted to be ~65 kDa, which is much smaller than the average CPD (~180 kDa) [45]. Known CPD sequences contain 3 active sites, which account for its extended length [44]; a feature that has been highly conserved through evolution, whereas only 1 active site is present in *A*. *queenslandica* CPD. This motif of 3 active sites is even present in basal CPs from choanoflagelletes species *Monosiga brevicollis* and *Salpingoeca rosetta*, disabusing the notion that the evolution of 3 active sites occurred after sponges, rather, suggesting that this difference is specific to sponges. It is likely that the reduced length of *Amphimedon* CPD contributes to its difficulty in phylogenetic classification. Interestingly, basal alga *C*. *reinhardtii* shows two ambiguous CPs, which also possess only one catalytic region but lack transmembrane regions [13], which are nonetheless clustered closest to basal CPDs by phylogenetic analysis (Fig 6). The unusual sequence structure of *Amphimedon* CPD warrants further investigation to fully understand the nature and function of this sequence, the exploration of other sponge CPs may provide interesting insights into its seemingly unusual evolution.

**Fig 7:**
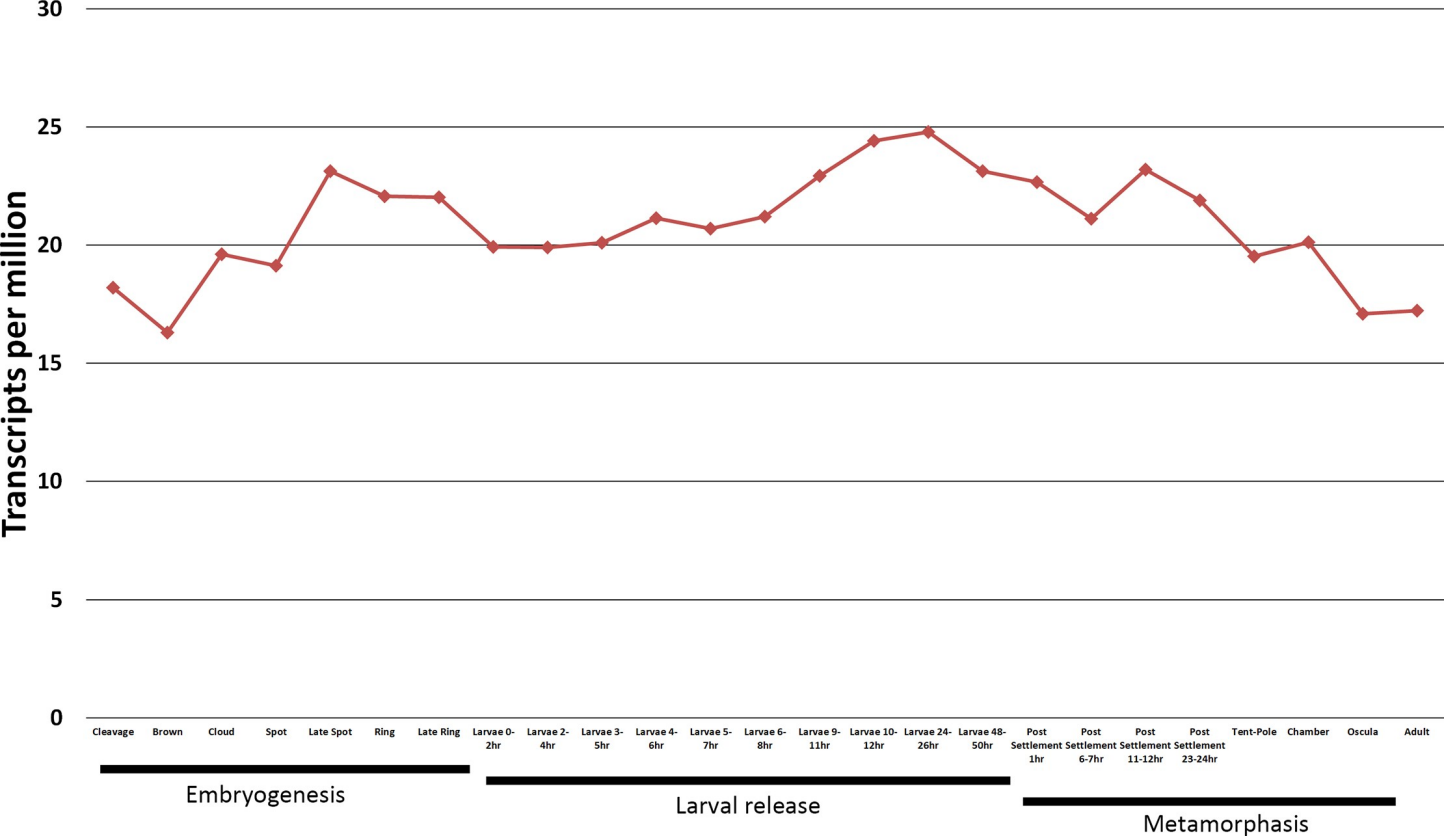
Gene expression of carboxypeptidases throughout life-stages of *A*. *queenslandica*. Stages are additionally grouped into 4 main phases; Embryo, Larvae, Metamorphosis and Adult.

*CPD* shows dynamic expression throughout *A*. *queenslandica* development, peaking at 24-26h for larval development, indicating an increased requirement during the larval phase (Fig 7).

## Conclusions

In this study, we have identified 12 genes that encode precursor processing components of *A*. *queenslandica*. Our phylogenetic analyses combined with domain analysis has helped to define several proteins into recognised categories of SP, PC or CP, and also demonstrates that some are sponge-specific and seemingly novel. We identify several features shared with fellow metazoan T. adhaerens, such as the absence of SP 22/23 as well as extensive furin-like repeats in PCs, suggesting developments specific to certain lower metazoans. Temporal gene expression analysis has refined our understanding of which peptidases are most utilised during development to adult, which may be targeted for further analysis. Included are the *A*. *queenslandica* SP18/21, furin and the unusual CPD, which show dynamic expression throughout the lifecycle of this sponge. Finally, NPC-A, which shows relatively high expression levels throughout development, could be further studied to understand their novel molecular mechanism of activity.

## Supporting information

S1 Fig ClustalW alignment of catalytic regions of 12 initial qualifying *Amphimedon queenslandica* prohormone convertases. Red arrows indicating critical D-H-S residues necessary for catalytic activity, as well as conserved N residue indicated by yellow arrow.
(TIF)

S1 File Protein sequences and accession numbers for all species sequences used for phylogenetic analysis. (TXT)
