# Supplementary figures and images for: Characterisation of early metazoan secretion through associated signal peptidase complex subunits, prohormone convertases and carboxypeptidases of the marine sponge (Amphimedon queenslandica)
Source: PLoS One. 2019 Nov 12;14(11):e0225227. doi: 10.1371/journal.pone.0225227 (PMC6850559; doi:10.1371/journal.pone.0225227)

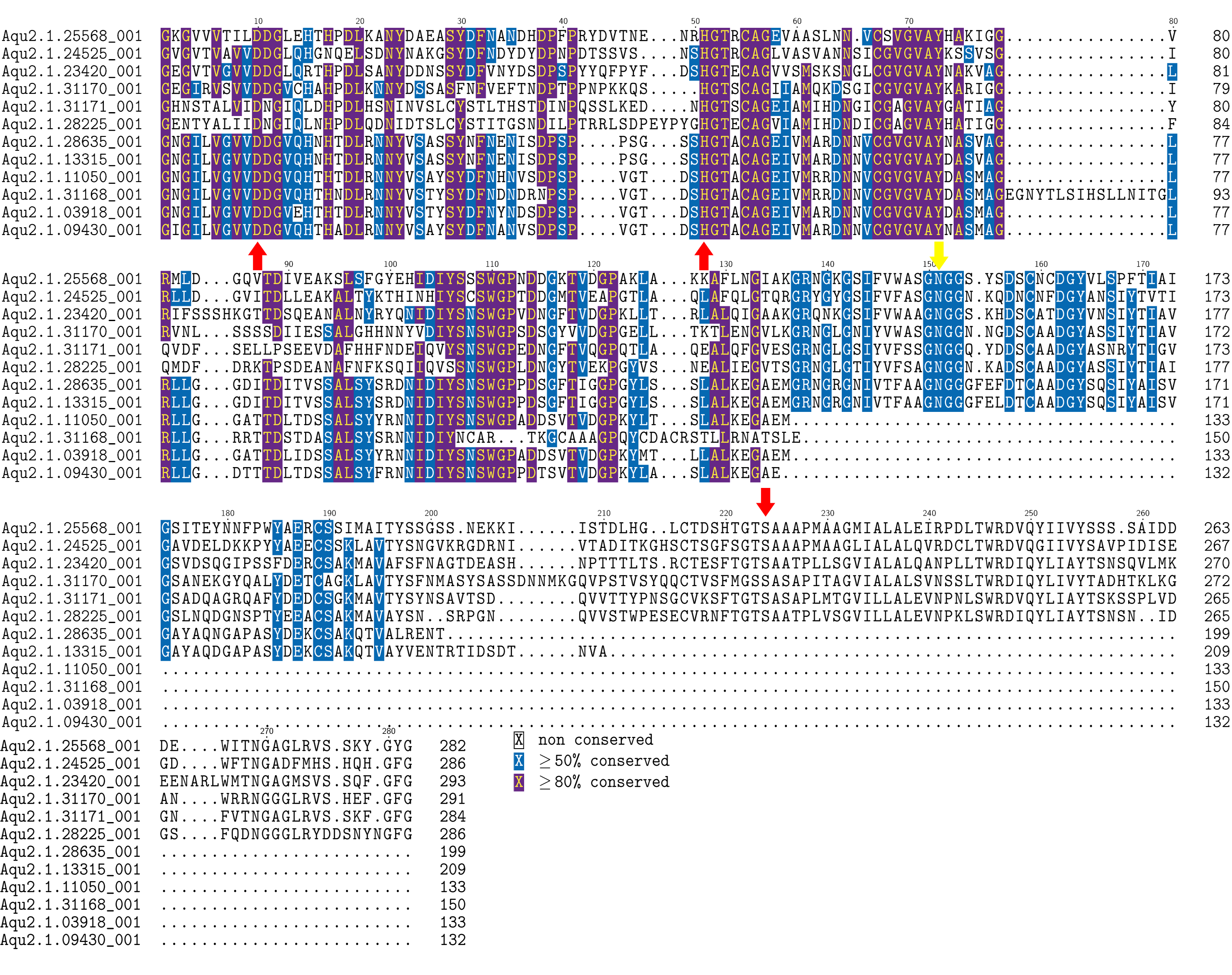

Supplement: S1 Fig — Red arrows indicating critical D-H-S residues necessary for catalytic activity, as well as conserved N residue indicated by yellow arrow. (TIF) [file pone.0225227.s001.tif]
